# A Low-Noise DC Seismic Accelerometer Based on a Combination of MET/MEMS Sensors

**DOI:** 10.3390/s150100365

**Published:** 2014-12-26

**Authors:** Alexander Neeshpapa, Alexander Antonov, Vadim Agafonov

**Affiliations:** 1 Center for Molecular Electronics, Moscow Institute of Physics and Technology, Moscow 117303, Russia; E-Mails: antonov.mipt@gmail.com (A.A.); agvadim@yandex.ru (V.A.); 2 R-sensors LLC, Dolgoprudny, Moscow Region 141700, Russia; 3 NordLab LLC, Dolgoprudny, Moscow Region 141700, Russia

**Keywords:** accelerometer, molecular-electronic transducer, combined sensor

## Abstract

Molecular-electronic transducers (MET) have a high conversion coefficient and low power consumption, and do not require precision mechanical components thus allowing the construction of cost- and power-efficient seismic accelerometers. Whereas the instrumental resolution of a MET accelerometer within the 0.1–100 Hz frequency range surpasses that of the best Micro-Electro Mechanical Systems (MEMS) and even some force-balanced accelerometers, the fundamental inability to register gravity or, in other words, zero frequency acceleration, significantly constrains the further spread of MET-based accelerometers. Ways of obviating this inherent zero frequency insensitivity within MET technology have so far, not been found. This article explores a possible approach to the construction of a hybrid seismic accelerometer combining the superb performance of a MET sensor in the middle and high frequency range with a conventional on chip MEMS accelerometer covering the lower frequencies and gravity. Though the frequency separation of a signal is widely used in various applications, the opposite task, *i.e.*, the combining of two signals with different bandwidths is less common. Based on theoretical research and the analysis of actual sensors' performance, the authors determined optimal parameters for building a hybrid sensor. Description and results for implementation of the hybrid sensor are given in the Experimental section of the article. Completing a MET sensor with a cost-effective MEMS permitted the construction of a low noise DC accelerometer preserving the noise performance of a MET sensing element. The work presented herein may prove useful in designing other combined sensors based on different technologies.

## 1. Introduction

Seismic accelerometers, along with seismometers, are the two widespread types of sensors used in seismology. Accelerometers are also commonly used in structure monitoring and seismic prospecting. In the field of weak signals like passive seismic exploration or response observation of signals induced by a nearby tower building or similar massive objects, the noise performance of a sensor should be foremost. With the lower noise sensor weaker signals can be distinguished from noise and regions further from the source can be covered.

Equipment in this field consists of rather expensive force-balanced accelerometers, which are quite delicate devices and therefore require qualified personnel to operate them at the point of observation. It should be pointed out that these devices have established an industry standard for seismic accelerometers with a sensitivity of several volts per *g* and a frequency range from DC to 100–200 Hz [1–3]. Another family of sensors potentially applicable in this field is that of Micro-Electro Mechanical Systems (MEMS) [4–7] which are less expensive, maintenance-free, with corresponding sensitivity and which overlap the desired frequency range.

Significant efforts are devoted to decrease the noise level of MEMS accelerometers. The most advanced of them are classified as “seismic grade” and have a noise level in the 50–300 ng/√Hz range [8,9]. In comparison with regular MEMS accelerometers the “seismic grade” ones are produced using more complicated bulk micromachined methods and are by far more expensive. Meanwhile the noise performance of the commercially available MEMS is still insufficient for weak seismic signals [10].

At the same time, molecular-electronic transducers (MET) allow for the design and production of both low noise velocimeters and accelerometers. The operation principle of MET sensors is based upon the deviation of an interelectrode current of the MET cell caused by the inertial movement of a liquid electrolyte encapsulated within a volume with elastic rubber bounders at both ends [11,12]. Modern MET sensors are of small size, highly shock resistant, provide high sensitivity and low noise. Accelerometers based on MET sensors are cost and energy effective and have much better noise performance [11], compared with the best commercially available Micro-Electro Mechanical Systems (MEMS) [7].

For a MET cell to be functional under a constant acceleration, it would require a continuous flow of electrolyte. However since the size of a cell and, hence, the volume of electrolyte is finite, the flow of electrolyte stops after all liquid collects on one side of the cell and the signal vanishes. This imaginary observation illustrates the two important features of a MET cell—on the one hand it is unable to register the zero frequency and on the other, providing the elastic rubber boundary is springy enough to compensate for the weight of electrolyte, it is self-aligned and stays operational under any tilt to the vertical.

The fundamental inability to register zero frequency acceleration constrains significantly the further spread of MET-based accelerometers. Since ways to obviate this inherent zero frequency insensitivity of a MET cell have so far not been found, we have endeavored to supplement a MET accelerometer with a DC component from a low-cost MEMS accelerometer. This approach allowed us to develop a hybrid accelerometer fully compliant with the above-mentioned industry standard which combines the full frequency range provided by the MEMS DC component while preserving the advantages of better noise, high cost and energy effectiveness of a MET sensor.

The DC component of an accelerometer may provide an opportunity to calibrate the sensor by rotating or tilting it at different frequencies and speeds. This signal can also be used to determine the sensor's axes orientation with respect to vertical. One of the important tasks to be solved during the integration of two sensors is preservation of phase integrity. This results in a sensor with amplitude-phase parameters identical or very close to those of common DC sensing accelerometers.

The technique of frequency separation of signal is widely used in various applications, from signal processing and analysis in scientific research and engineering solutions to image sharpening and audio playback. On the other hand, the opposite technique, *i.e.*, combining two signals with different bandwidths, is less common, although it may benefit the combined sensor performance over that of the original ones separately [13]. The theoretical section of this paper describes the method of combining signals from two sensors of different types in order to achieve optimal resolution and bandwidth. It may refer to any combination of sensors where one has better performance within a particular frequency band and the other performs better outside this region. The practical part of the article gives description and results for implementation of the combined DC accelerometer comprising a MET and a MEMS sensor.

The approach outlined in this article, however, does not depend on the sensor type; it can be achieved ether by means of analog circuitry of by digital signal processing, so this work may prove useful for any possible array of different types of sensors selected to achieve optimal performance by their combination.

## 2. Experimental Section

### 2.1. Theory

The method for combining the sensor's signals implies proper filtering of each signal to achieve the predefined frequency response and further summation of the signals so that the signal from one sensor covers a low-frequency region, and the signal from other sensor a high frequency region. We specify our frequency range of interest to be within the pass bands of both low-pass and high pass filter at a level of −40 dB of the pass band level. Having defined the area of intersection this way, we can put aside the sensors' mutual influence outside this area since the signal from a sensor in the stop band is reduced to a negligible 1% or less. In order to obtain flat frequency response in the area of intersection of two sensors the following relation should be satisfied:

(1) $$At_{S1}\;p_{S1}\;+Bt_{S2}\;p_{S2}=C$$

here A, B and C are gain coefficient constants, *t_Sn_*—frequency responses of sensor 1 and sensor 2 expressed in terms of transfer functions and *p_Sn_*—transfer functions of the filter applied to corresponding sensor's signal. Further, for simplicity, we shall assume that in the area of intersection of the sensors their frequency responses are flat so *t_Sn_* = 1. In addition, we also assume that both sensors have the desired sensitivity and all transfer functions are normalized so that A, B, and C coefficients are equal to each other and can be cancelled:

(2) $$p_{S1}+p_{S2}=1$$

The simplest system, which clearly represents this relation, consists of two first-order basic RC filters—that is when one signal is passed through a low-pass filter and other is passed through a high-pass filter of the same cut off frequency, the resulting transfer function appears to be equal to 1:

(3) $$\frac{1}{1+\text{if}\tau }+\frac{\text{if}\tau }{1+i\tau }=1$$

(4) $$\tau =\frac{1}{f_{c}}=RC$$

where *f_C_* is the cut off frequency and *τ* is the time constant for the filters.

Obviously, in this case the spectrum of the output signal consists of a low-frequency part of the signal from the first sensor and a high-frequency part of the signal from the second sensor while the whole system maintains flat amplitude and zero phase frequency response regardless of the cut off frequency *f_c_*. These considerations are valid for filters of any order and complexity until the relation (2) is satisfied. The precise form of the transfer functions in this case depends on the initial frequency response of the sensors, desired noise performance and possibility of practical implementation of the required filters.

We study a model where the sensor S1 is assumed to have better performance at low frequencies while the sensor S2, on the contrary, performs better at higher frequencies. To assure a better separation of the S1 sensor's signal at higher frequencies, its signal is filtered by two consecutive 1st order filters. In particular, this situation describes a combination of a MEMS (for S1) and a MET (for S2) linear accelerometers:

(5) $$p_{S1}=p_{\text{MEMS}}={\left(\frac{1}{1+\text{if}\tau }\right)}^{2}$$

(6) $$p_{S2}=p_{\text{MET}}=1-p_{\text{MEMS}}=\frac{\text{if}\tau }{1+if\tau }\frac{2+if\tau }{1+if\tau }$$

**Figure 1.**
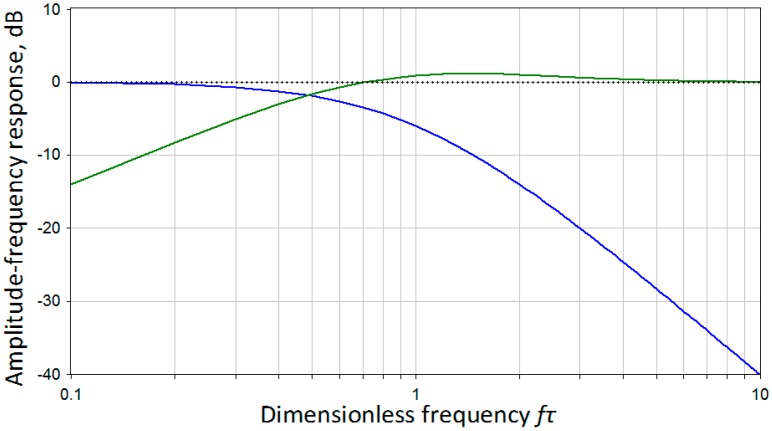
Amplitude-frequency response of transfer functions (2)—blue, (3)—green and their sum—black dotted.

The implementation of the filters (5) and (6) is relatively simple and can be carried out by means of analog circuitry. On the other hand, using a second-order filter for the S1 sensor allows more efficient noise reduction at higher frequencies compare with the first-order filtering developed in [13]. Finally, the transfer functions satisfy relation (2), *i.e.*, the resulting frequency response is flat. Figure 1 represents amplitude-frequency response for the transfer functions (5), (6) and for their sum, which is by definition equal to 1. For a detailed description of how these particular filters were implemented in hardware please refer to the later subsection of this paper.

**Figure 2.**
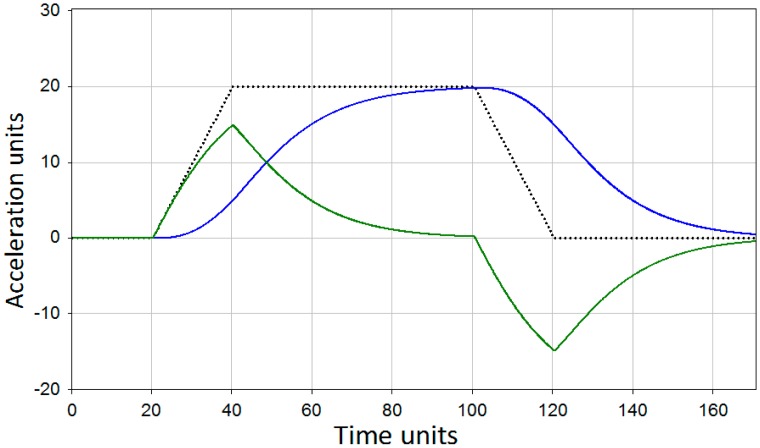
Time-domain simulation of the signal summation. Signal passed through the filter (2)—green line, signal passed through the filter (3)—blue line. Resulting signal—black dotted line.

Figure 2 represents a simple visual simulation of how the described method operates in the time domain. This demonstration shows how the functions (5) and (6) act on an input signal starting from zero value, linearly increasing to some constant and later linearly decreasing to zero again. It can be seen that after a summation the correct form of the signal will be restored.

The expressions (5) and (6) contain a parameter *τ*, which is the time constant. This parameter defines the position of the boundary between two sensors frequency ranges. The alteration of the parameter ***τ*** affects the overall noise performance of the system and serves as a means of optimizing it. The optimal boundary frequency selection should be based on the comparative analysis of the sensor's noise characteristics. This analysis can be performed in terms of the noise spectrum density.

It is important to note that sensor's self-noise signals are considered to be stochastic. This circumstance makes them behave differently under summation operation compared to real signals. Since the sensor's noises are uncorrelated, the output noise level distribution is given by a following expression:

(7) $$e_{r}(f)=\sqrt{{\left(e_{S1}\;(f)\left|p_{S1}\;\right|\right)}^{2}+{\left(e_{S2}\;(f)\left|p_{S2}\right|\right)}^{2}}$$

where *e_Sn_*(*f*)—sensors' self-noise spectrum distribution, *e_r_*(*f*) —output noise spectrum distribution of the system.

In other words the total noise of a combined system depends on both self-noise levels of the two sensors multiplied by magnitudes of corresponding filter's transfer functions. Solving this equation gives the output noise performance of a combined system for given sensors' noise models *e_S_*(*f*).

Considering our particular system we use the following simplified noise models: the MEMS noise is known to be “white”, *i.e.*, has flat noise spectrum distribution [6]. The level of MEMS' noise is assumed to be about −60 dB. Since the sensitivity of a MET sensor falls as it approaches zero frequency, its noise level rises and eventually intersects with the MEMS' noise level. At the region of the noises' intersection we assume the MET noise to adhere to the “pink” noise pattern, *i.e.*, to have an ∝ 1/*f* spectrum distribution. At higher frequencies the noise flattens at the level about −110 dB. This model is consistent with the theoretical and experimental results obtained in the papers [14,15]. The lower-frequency “pink” noise corresponds to the “geometrical” noise, and the higher-frequency “white” noise—to the “thermal” noise of a MET cell. The frequency *f_i_* that corresponds to a point of intersection of sensors' noise levels plays a decisive role in selecting the filter parameters.

**Figure 3.**
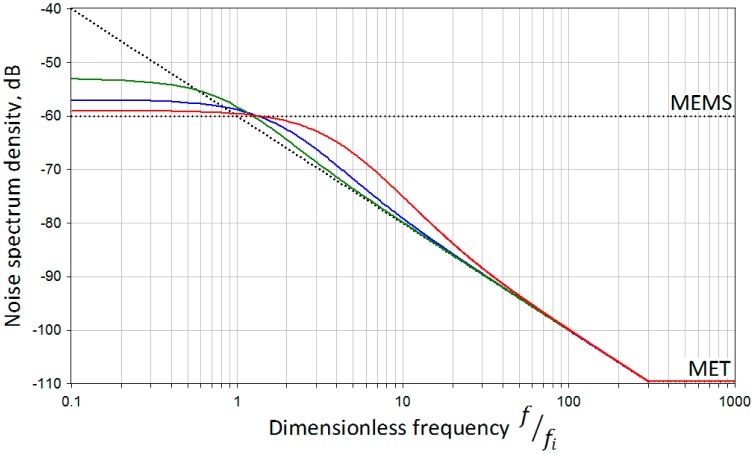
Output noise distribution pattern depending on parameter τ. Black dotted—MET and MEMS noise models; Green—output noise when τ = 1; Blue—output noise when τ = 0.5; Red output noise when τ = 0.25.

Figure 3 shows the two above mentioned noise distribution patterns and the behavior of their summation depending on the *τ* parameter, where *τ* = 1/*f_c_* or (*f_i_* = *f_c_*) for the green line; *τ* = 1/2*f_c_* or (*f_i_* = 2 · *f_c_*) for the blue line and *τ* = 1/4*f_c_* or (*f_i_* = 4 · *f_c_*) for the red line.

Dotted lines represent the assumed initial noise levels for MET and MEMS. Here for simplicity we plot the abscissa axis in dimensionless coordinate f_c_/f_i_ so that the point of intersection of sensor's noises would be at the value of 1 unit. It can be seen that the red pattern introduces the smallest addition to the original MEMS noise at lower frequencies while compromising the overall noise performance in the area of intersection. As an alternative, the green pattern keeps the noise in the area of intersection close to optimal, while impairs the low frequency performance of a system. As it is shown in the later subsection, the actual noise curve of a MET sensor rises slower than 1/f when approaching lower frequencies, so the green pattern or (f_i_ = f_c_) might prove a better selection for our case.

### 2.2. Experimental Implementation

For the purpose of testing the technique and theoretical calculations, a pair of accelerometers of different type was chosen. A molecular-electronic accelerometer MTSS-1043A with a standard passband of 0.1 Hz–120 Hz and 70 ng/Hz^½^ noise density at 10 Hz [16] played the role of a better performing but limited passband sensor. The signal from the MET sensor was complimented in lower frequencies by adding the signal from an Analog Devices ADXL103 MEMS accelerometer. Though the MEMS accelerometer has the lowest noise density of 110 μg/Hz^½^ among AD analog accelerometers [6], its noise level is more than 1000 times greater than that of the MET at 10 Hz.

**Figure 4.**
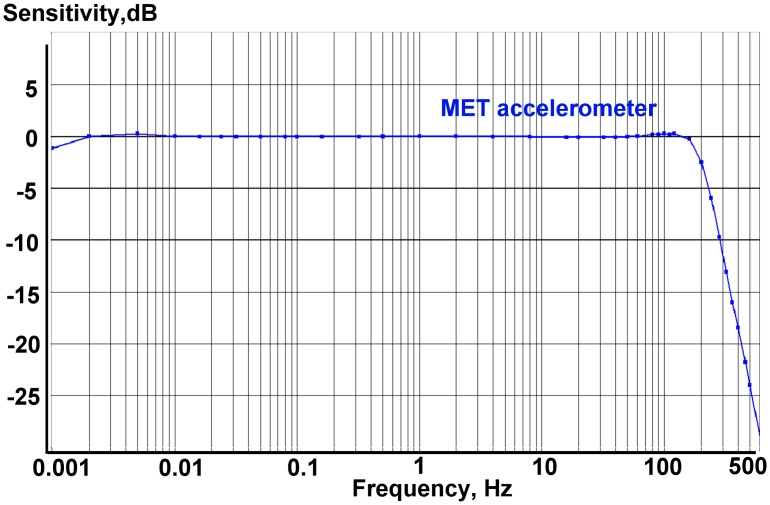
The extended low frequency MET sensor amplitude response. No output filters applied. $0\;\text{dB}=1\overset{V}{\;}/\underset{g}{\;}$.

The first step in assessing the parameters of the system was to obtain the noise plot for the MET sensor in the lowest frequencies (LF) range. For that purpose the standard sensor parameters were modified in order to extend its passband to the lowest frequency possible. The resulting sensitivity chart was accurately measured down to 0.001 Hz (1000 s). The frequency response was taken with use of the accelerometer feedback cascade coil [11]. As it can be seen on the Figure 4, the LF cut-off by −3 dB level had thus been extended to 1000 s.

For the noise performance study, a long term record of the sensor's signal was taken. Considering the recorded data from that point on, all signal records were taken under the same conditions—the sensors were installed on a thick granite plate in a vault of the office building. All recordings were made at night during 1–2 h of the quietest period. The low-noise force feedback molecular-electronic seismometer CME-6211 [17], which was installed on the same plate, was used for monitoring the ambient signals. This seismometer has sensitivity of 2000 V/(m/s) within 0.0167 Hz (60 s)–50 Hz passband and self-noise level of −160 dB in the range of interest, which is at least 50 dB below the noise level of the MTSS1043A.

**Figure 5.**
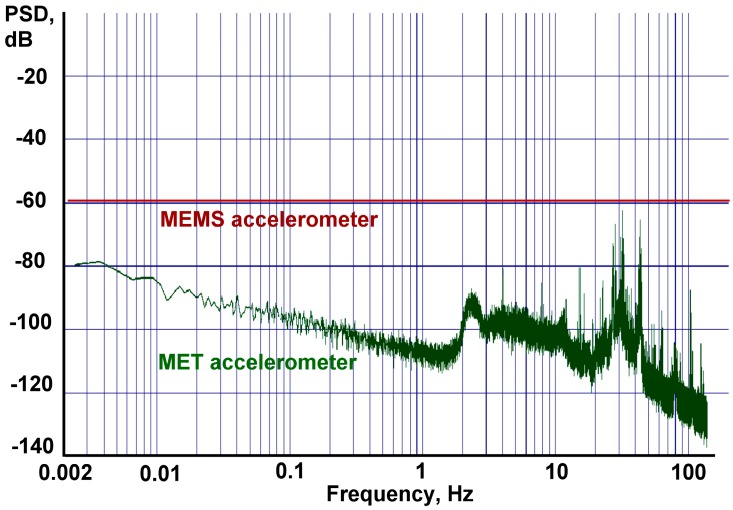
Noise power spectral density (PSD) comparison, $0\;\text{dB}=1\frac{m}{S^{2}\sqrt{Hz}}$.

The actual ground motion observed by the seismometer in the frequency range from 2 Hz and lower lies significantly below the accelerometers' signal, so for our region of interest which is below 1 Hz, the output of an accelerometer is presented by the sensor's self-noise only. Data were recorded by a DAS-6101 16-channel 22-bit acquisition system at 320 samples per second. The noise performance comparison between measured values for the extended LF MET accelerometer and datasheet level for the MEMS accelerometer is given in Figure 5.

As we can see, the noise performance of the ADXL103, which is defined by the manufacturer as 110 μg/Hz^½^ or, in units used on our plot, as −59.2 dB, goes much higher than the noise of the MET accelerometer. The noise density of the latter, in turns, is rising as the frequency gets lower. This noise behavior is close to that examined in the previous section (see Figure 3, where one sensor has a uniform noise plot and the other a rising noise plot towards the zero frequency, though the dependence in the range of interest is not completely linear as it was in the model, otherwise there should be an increase by 20 dB per decade at lower frequencies).

From the presented graphic data we estimate that the noise curves intersection occurs at about 0.0001 Hz of even further in the LF range and that the intersection frequency *f_i_* is at least equal or even lower than that value. Considering the filter parameters, and keeping in mind that for the optimal noise performance the cut off frequency should be between *f_i_* and 4*f_i_*, we see that the MEMS low pass filter cut off frequency *f_c_* is between 0.0001 Hz and 0.0004 Hz. Having the intersection frequency as low as that might not only hamper further implementation of the circuitry, but also complicates the experimental validation of amplitude-phase parameters at such low frequencies. For the purpose of testing the technique we decided to raise the low pass cut off frequency *f_c_* to 0.02 Hz. Although not being optimal in terms of the noise performance, this decision facilitates noise observation and frequency response measurements.

**Figure 6.**
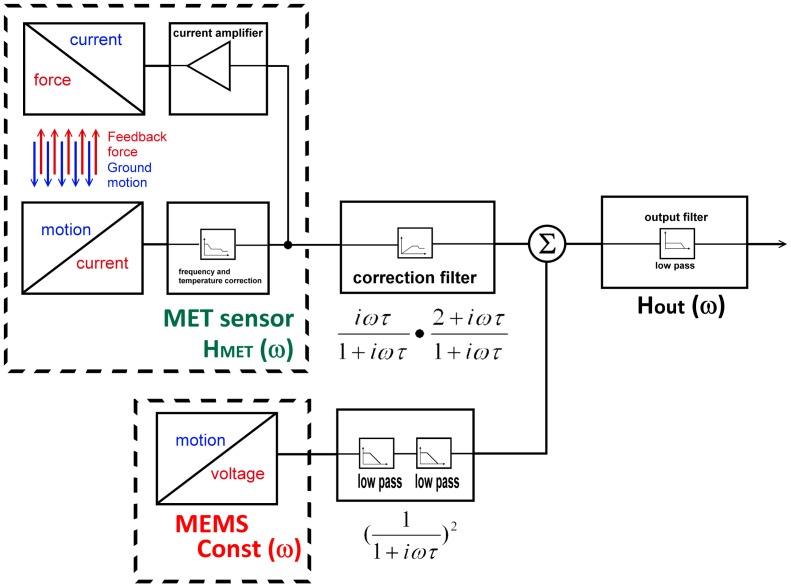
Block diagram for a combined sensor.

Figure 6 represents the block diagram for a combined sensor. Sensors in the dashed boxes are the MET and MEMS, correspondingly. However, for the purpose of combining the principle of operation of sensors, this is of no significant importance. The only two conditions which should be upheld are that the sensitivities of both sensors are equal and that the output responses within the range of interest are flat. The evenness of the MEMS sensor is guaranteed by the manufacturer, while the evenness of the MET sensor in the combination range is confirmed by the amplitude response curve presented in Figure 4. The adjustment of the MET sensors sensitivity to the MEMS sensor sensitivity is achieved by varying the gain of the feedback current amplifier. The final check of the conversion coefficient of the two sensors was made by the simultaneous recording of the tilt signals on a shaking table.

**Figure 7.**
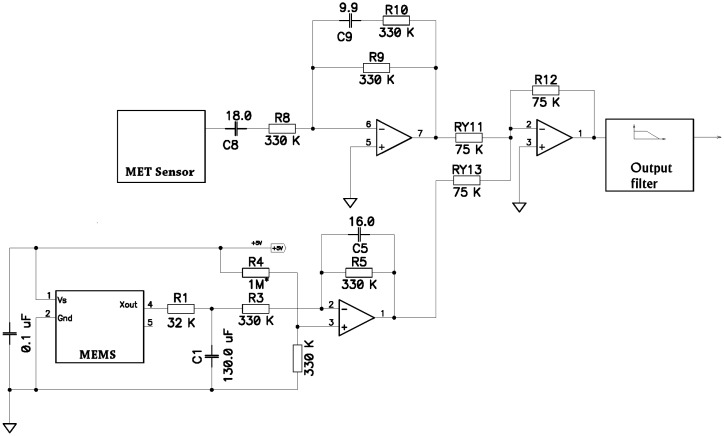
Schematic diagram for the combined filters.

The MEMS filter consists of two consecutive 1st order low-pass filters while the MET filter is a combination of a 1st order high-pass filter and a step-like low-pass filter. After passing the filters, the two signals are combined in the adder and pass through the 2nd order output low pass filter, which forms the desired high frequency response. Figure 7 represents the schematic realization of the above block diagram.

To estimate the effect of the addition of the three amplifier stages on noise performance we consider the noise performance of an AD704 quad operational amplifier [18], which was used in this circuitry. Since the transfer constant of the output filter in the passband is pretty close to 1, we can compare the noise voltage introduced by a single OP-amp stage by converting it to the acceleration at input with use of 1 V/g or 10 V/(m/s^2^) conversion coefficients, which correspond to original sensors' sensitivity. For the given by the manufacturer values of equivalent input voltage noise density *Vn* = 17 nV/Hz^½^ and input current noise density *I_n_* = 50 *fA*/Hz^½^, we can calculate the noise of a single stage at 10 Hz as $U_{n}=\sqrt{V_{n}^{2}+{\left(I_{n}\cdot R9\right)}^{2}}$, where R9 is a 330 kΩ feedback resistor. The calculation shows that the input voltage noise gives the major contribution into the output noise and *U_n_* = 23.7 nV/Hz^½^ or 2.37 ng/Hz^½^ or −152 dB according to the vertical axis units used in Figure 5. Therefore, the addition of several stages will not affect the overall sensor's noise performance.

**Figure 8.**
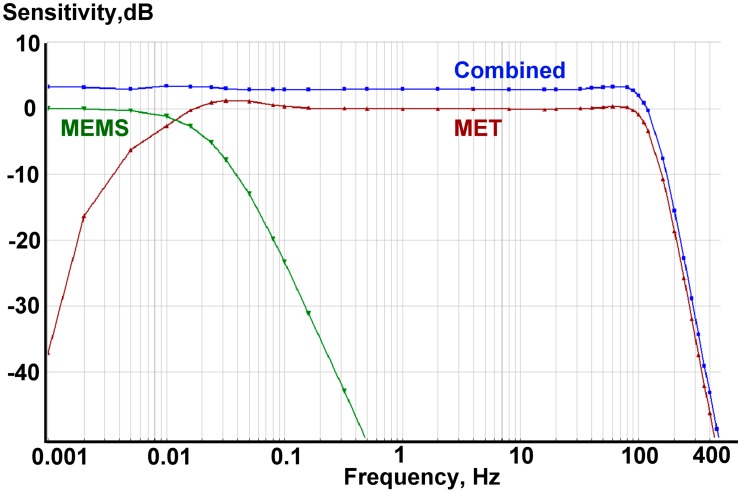
Simulated amplitude response. Overall system response (blue) is raised by +3 dB for better visualization. Red is a filtered MET sensor response, while green is that for MEMS.

**Figure 9.**
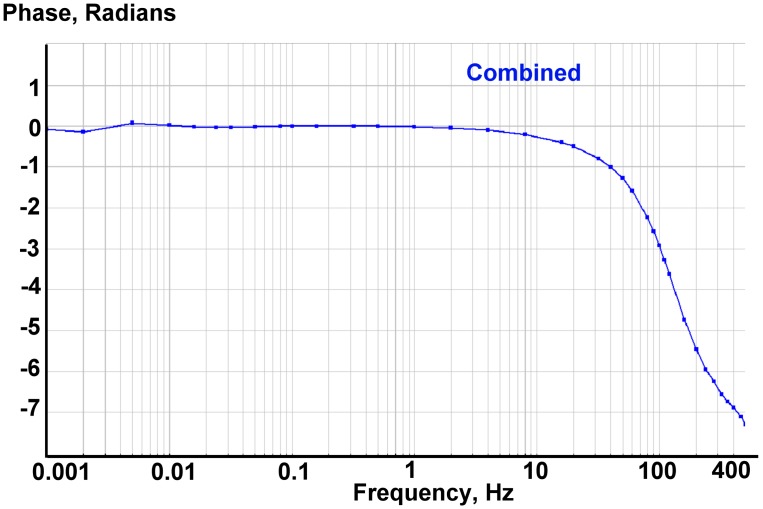
Simulated overall system phase response.

The frequency response of the combined system was simulated in the DADiSP^®^ program. The frequency response for a MET was taken from the measurements for the specific MET sensor, while the response for MEMS was assumed to be flat and zero phased. The results for the computer simulation are given on Figures 8 and 9.

**Figure 10.**
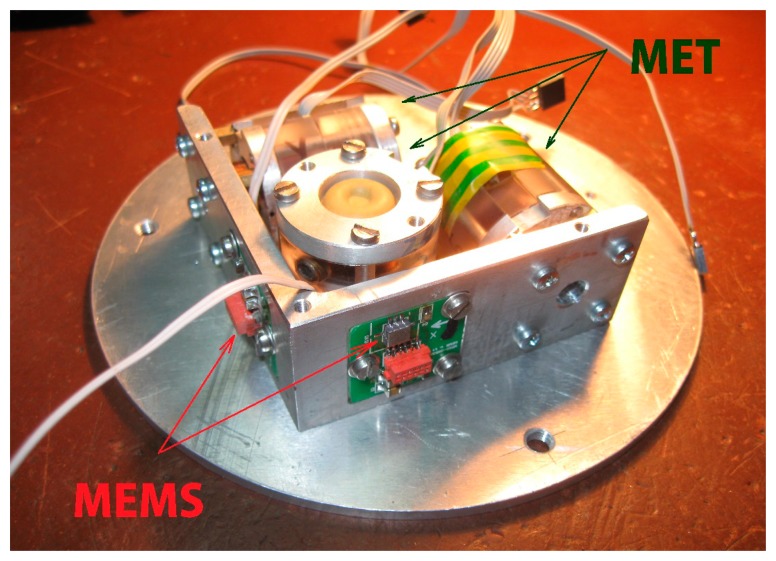
Sensing elements of the combined sensor.

The final tests were made with use of a 3-axes combined DC accelerometer that was built of three similar MET sensors installed orthogonally and two MEMS sensors, one of which was two-component ADXL 203 and another—one component ADXL 103 installed in the way they could form three orthogonal axes (see Figure 10).

**Figure 11.**
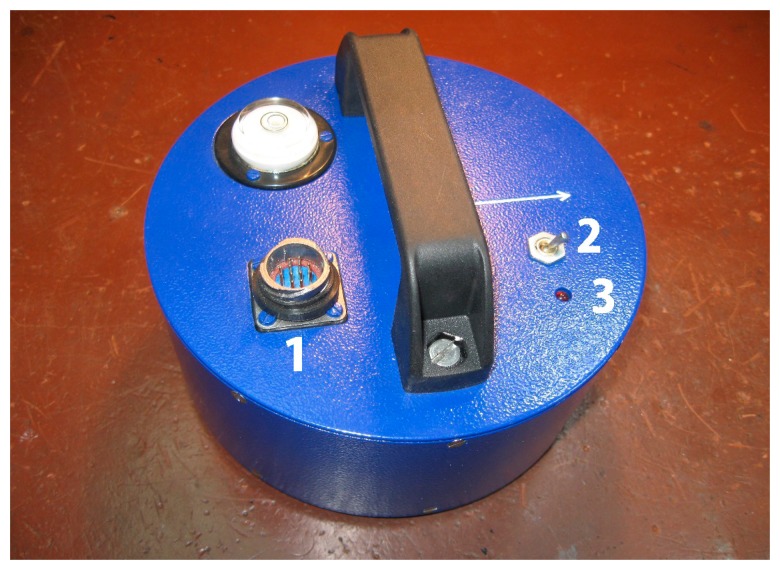
Combined sensor. Marked are output connector (1), MEMS switch (2), MEMS state indicator (3).

The whole assembly was placed in a hermetical case. The switch installed on the front panel allowed turning on and off the addition of a MEMS signal, so the recording of the two modes of operation—a fully combined and a MET sensor only—could be performed without any discontinuity (see Figure 11).

**Figure 12.**
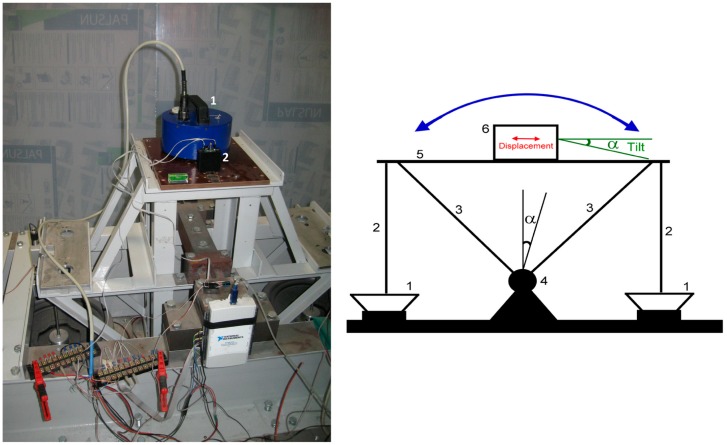
Low frequency response calibration. Marked on the left side are the hybrid accelerometer (1) and the MEMS on a pedestal (2).

The experimental measurements of the hybrid sensor response were done using the shake table. Our interest is in the measurement of the response at very low frequencies, while the majority of shake-tables are designed for operation at much higher frequencies. Indeed, to induce an acceleration of 1 mm/s^2^ at 0.01 Hz, which corresponds to 100 mV output signal, the linear displacement should be about 25 cm. In case of a tilt, the same acceleration can be easily induced by an inclination by mere 0.57 degrees from vertical regardless of the frequency. That's why we used a tilting calibration platform designed for calibration of the broad-band seismometer and implemented in LLC “R-sensors” laboratory. A scheme and actual view of the platform are shown on Figure 12, right and left, respectively. The construction of the shaking table allows the horizontal plate (5) which is installed on two brackets (3) to execute a rotational motion around a pivot (4) under the exertion generated by two loudspeakers (1) transferred via shafts (2). The linear displacement of each shaft is measured by two precise displacement sensors (not shown in the figure). The operating principles and practical implementation of such a shake-table are described in [19].

**Figure 13.**
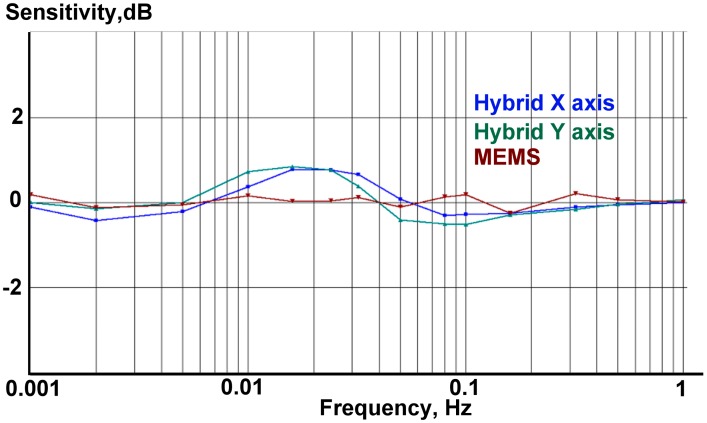
Measured amplitude response for two horizontal channels of the hybrid sensor (blue and teal) and a MEMS (dark red). $0\;\text{dB}=1\overset{V}{\;}/\underset{g}{\;}$.

**Figure 14.**
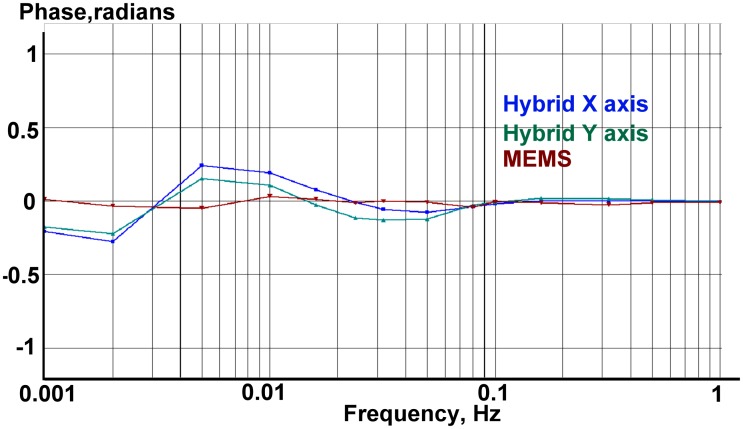
Measured phase response for two horizontal channels of the hybrid sensor (blue and teal) and a MEMS (dark red).

The accelerometer under test was placed on the calibration platform with the sensitivity axis aligned horizontally. Next to the hybrid sensor an ordinary ADXL103 MEMS sensor was installed in order to verify the data and later allow comparison of the AFCs. The multichannel 16-bit analog to digital converter of the multifunction data acquisition NI-6218 [20] was used for collecting the signal from shake table reference sensors and the output signal of the accelerometers. The results for the measured low frequency amplitude and phase response of the combined sensor are given in Figures 13 and 14, correspondingly.

**Figure 15.**
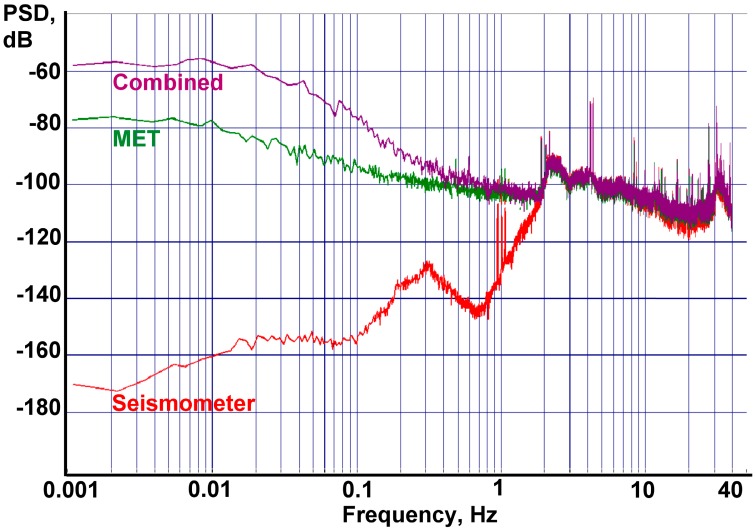
Final PSD. Purple is the combined sensor, green is the MET only sensor with the correction filter, red is the reference seismometer CME-6211. $0\;\text{dB}=1\frac{m}{s^{2}\sqrt{Hz}}$.

The spectral density of the final recording is presented in Figure 15. Data were recorded by a DAS-6101 16-channel 22-bit acquisition system at 80 samples per second.

Figure 15 shows that according to the seismometer, the ground motion signal lies beneath the output signal of both accelerometers from 2 Hz to the lower frequencies. The noise of the combined sensor corresponds to the MEMS noise level at frequencies lower than 100 s, while from 1 Hz and higher it corresponds to the MET sensor level. This behavior fully complies with the one predicted in the theoretical part.

## 3. Conclusions

A method for combining two sensors of different types is presented. In this approach the signal from one sensor covers a low-frequency region, and the signal from other sensor a high frequency region, while preserving flat amplitude and zero phase response. The method could be used to achieve a combined sensor with better overall performance of either of the original sensors separately. The technique was implemented in a combined molecular-electronic (MET)/MEMS accelerometer. The overall unevenness of the measured response characteristic in the range of signals intersection stays well within ±0.7 dB, while the phase response stays close to zero. Compared to the conventional MET accelerometers this approach allows for extending the frequency range to DC, while keeping the self- noise several orders of magnitude below the self-noise of the MEMS. The result of the practical implementation strongly depends on the sensor parameters and the methods used for combining their responses. Though higher order filters may give better results, hardware implementation by means of analog circuitry could be rather difficult. For the case of 3rd and higher order filters digital signal processing might be the most practical means for realization of this technique.

The suggested method can be developed further by selecting a less noisy MEMS like that described in [7] or by implementing a digitally combined system where both signals are digitized independently by a separate analog to digital converter and after a digital processing are combined into one output data stream using optimal filtering procedures.

## References

[1] Sercel 508XT Brochure (English) http://www.sercel.com/products/Lists/ProductSpecification/508XT_brochure_Sercel.pdf.

[2] Kinemetrics EpiSensor ES-T Force Balance Accelerometer Datasheet. http://www.kinemetrics.com/uploads/PDFs/ES-T%20Datasheet.pdf.

[3] CMG-5T Strong Motion Feedback Accelerometer. http://www.guralp.com/documents/DAS-050-0001.pdf.

[4] Colibrys SF1600 and SF2006 Application Note Seismic Accelerometers for Unattended Ground Sensors (UGS). http://www.ims-i.za.com/pdf/30N.UGS.B.05.11.pdf.

[5] Colibrys Si-FlexTM Series Product Description. http://www.colibrys.com/files/pdf/products/PD%20SiFlex%2030D.SFX.D.03.09.pdf.

[6] Analog Devices ADXL103/ADXL203 Datasheet. http://www.analog.com/static/imported-files/data_sheets/ADXL103_203.pdf.

[7] Silicon Design Inc Low Noise High Stability Analog Surface Mount Accelerometer Model 1521. http://www.silicondesigns.com/pdfs/2012.pdf.

[8] Yamane D., Konishi T., Matsushima T., Machida K., Toshiyoshi H., Masu K. (2014). Design of sub-1g microelectromechanical systems accelerometers. Appl. Phys. Lett..

[9] Homeijer B., Lazaroff D., Milligan D., Alley R., Wu J., Szepesi M., Bicknell B., Zhang Z. Hewlett Packard's Seismic Grade Mems Accelerometer.

[10] Aizawa T., Kimura T., Matsuoka T., Takeda T., Asano Y. (2008). Application of MEMS accelerometer to geophysics. Int. J. JCRM.

[11] Agafonov V.M., Egorov I.V., Shabalina A.S. (2014). Operating principles and technical characteristics of a small-sized molecular-electronic seismic sensor with negative feedback. Seism. Instrum..

[12] Huang H., Agafonov V., Yu H. (2013). Molecular Electric Transducers as Motion Sensors: A Review. Sensors.

[13] Westhora K. ELF Extended Low Frequency Sensor Designs.

[14] Agafonov V.M., Zaitsev D.L. (2010). Convective noise in molecular electronic transducers of diffusion type. Tech. Phys..

[15] Zaitsev D.L., Dudkin P.V., Agafonov V.M. (2006). Fluctuating vortex flows and their contribution to the noise of molecular electronic converters. Izv. Vyssh. Uchebn. Zaved. Electron..

[16] Compact Molecular-Electronic Seismic Sensors. http://r-sensors.ru/1_products/Compact_seismic_sensors_MTSS.pdf.

[17] Broadband Seismometer CME-6211. http://r-sensors.ru/1_products/Descriptions/CME-6211.pdf.

[18] AD704: Picoampere Input Current Quad Bipolar Op Amp. http://www.analog.com/static/imported-files/data_sheets/AD704.pdf.

[19] Abramovich I., Agafonov V., Daragan S., Kazak B. (1996). Wide Band Motion Sensor Calibrator. Seismol. Res. Lett..

[20] Bus-Powered M Series Multifunction DAQ for USB–16-Bit. http://www.ni.com/datasheet/pdf/en/ds-9.

